# High-flow oxygen therapy through nasal cannulae versus low-flow oxygen therapy via Venturi mask after extubation in adult, critically ill patients

**DOI:** 10.1186/cc9585

**Published:** 2011-03-11

**Authors:** F Antonicelli, A Cataldo, R Festa, F Idone, A Moccaldo, M Antonelli, SM Maggiore

**Affiliations:** 1 'A. Gemell' University Hospital, Rome, Italy

## Introduction

Oxygen therapy, usually delivered with the Venturi mask, is frequently used in critically ill patients after extubation. This device delivers low-flow oxygen with cold humidification. Recently available is a new device for oxygen therapy through nasal cannulae (NHF). Such a device delivers up to 60 l/minute oxygen, with heated humidification. The aim of this study was to compare the effects of these two devices for oxygen therapy on arterial blood gases, discomfort and adverse events in critically ill patients after extubation.

## Methods

Inclusion criteria were mechanical ventilation for more than 24 hours and a successful spontaneous breathing trial with PaO_2_/FiO_2 _< 300 at the end of the trial. Exclusion criteria were tracheostomy, age <18 and anticipated need for non-invasive ventilation after extubation. Patients were randomized to receive oxygen therapy with NHF or Venturi mask after extubation. With both devices, nominal FiO_2 _was set to obtain SpO_2 _between 92 and 98% (between 88 and 95% in hypercapnic patients). Arterial blood gas, respiratory rate, and discomfort were recorded at 1, 3, 6, 12, 24, 36, and 48 hours from inclusion. Discomfort was assessed by asking patients to rate their discomfort related to the interface and to the upper airway dryness (mouth, throat, and nose dryness, difficulty to swallow and throat pain), using a numerical scale from 0 (no discomfort) to 10 (maximum discomfort).

## Results

Seventy-five patients were enrolled (40 NHF, 35 Venturi mask). No difference was observed in the baseline characteristics at inclusion. PaO_2_/FiO_2 _was higher in the NHF group, being statistically significant at 1, 3, 24, and 36 hours (317 ± 78 vs. 253 ± 84 at 24 hours, *P *< 0.01). PaCO_2 _was similar in the two groups. Nominal FiO_2 _and the respiratory rate were always lower with NHF than with Venturi mask (30 ± 6 vs. 37 ± 10%, *P *= 0.01, and 21 ± 4 vs. 27 ± 4 breaths/minute at 24 hours, *P *< 0.01, respectively). Discomfort due to the interface was higher with the Venturi mask at 12, 24, 36, and 48 hours (4 ± 3 vs. 6 ± 3 at 24 hours, *P *< 0.01). Discomfort related to dryness of the upper airways was also higher with the Venturi mask than with NHF at all time steps (2 ± 2 vs. 4 ± 2 at 24 hours, *P *< 0.01). Oxygen desaturations and interface displacements were more frequent with the Venturi mask than with NHF (94 vs. 40% patients, *P *< 0.01, and 71 vs. 30% patients, *P *< 0.01, respectively).

## Conclusions

NHF is an effective method for delivering oxygen therapy after extubation, allowing better oxygenation with less patient discomfort and adverse events than the Venturi mask.

